# A Prospective Observational Study of Physical Activity Levels and Physical Fitness of People at High Risk for Lung Cancer

**DOI:** 10.1016/j.jtocrr.2024.100633

**Published:** 2024-01-13

**Authors:** Asha Bonney, Catherine L. Granger, Daniel Steinfort, Henry M. Marshall, Emily Stone, Annette McWilliams, Fraser Brims, Paul Fogarty, Linda Lin, Jiashi Li, Siyuan Pang, Stephen Lam, Kwun M. Fong, Renee Manser

**Affiliations:** a Department of Medicine, University of Melbourne, Melbourne, Australia; b Department of Respiratory and Sleep Medicine, Royal Melbourne Hospital, Melbourne, Australia; c Department of Physiotherapy, Royal Melbourne Hospital, Melbourne, Australia; d Department of Physiotherapy, University of Melbourne, Melbourne, Australia; e Thoracic Research Centre, University of Queensland, Queensland, Australia; f Department of Thoracic Medicine, The Prince Charles Hospital, Chermside, Queensland, Australia; g Department of Thoracic Medicine and Lung Transplantation, St Vincent’s Hospital Sydney and School of Clinical Medicine UNSW Sydney, Sydney, Australia; h Department of Respiratory Medicine, Fiona Stanley Hospital, Murdoch, Western Australia, Australia; i Faculty of Health and Medical Sciences, University of Western Australia, Western Australia, Australia; j Department of Respiratory Medicine, Sir Charles Gairdner Hospital, Nedlands, Western Australia, Australia; k Respiratory Department, Epworth Eastern Hospital, Box Hill, Victoria, Australia; l Department of Medicine, The University of British Columbia, Vancouver, British Columbia, Canada

**Keywords:** Fitness, Lung cancer, Physical activity, Screening

## Abstract

**Introduction:**

Physical activity (PA) is a potentially modifiable risk factor for lung cancer, with previous research revealing that people who engage in more PA have lower risk of developing lung cancer. PA levels of lung cancer screening participants have not previously been explored.

**Methods:**

Participants at a single Australian International Lung Screen Trial site were eligible for assessment of self-reported PA levels (International Physical Activity Questionnaire and Physical Activity Scale for the Elderly) and physical assessments (6-min walk distance, hand grip muscle strength, daily step count, and body composition) at a single time point during lung cancer screening. Statistics were predominantly descriptive, with parametric data presented as mean and SD and nonparametric data presented as median and interquartile range (IQR).

**Results:**

A total of 178 participants were enrolled in this study, with a median age of 61 years. Of the participants, 61% were men and 51% were people who currently smoke. The median total International Physical Activity Questionnaire score was 1756 MET/min/wk (IQR 689, 4049). Mean total Physical Activity Scale for the Elderly score was 160 (SD 72), higher than described in healthy sedentary adults. The median daily step count was 7237 steps (IQR 5353, 10,038) and mean 6-minute walk distance was 545 m (SD 92). Median grip strengths were within predicted normal range, with an elevated median percentage body fat and low skeletal muscle mass found on body composition.

**Conclusion:**

Almost a quarter of International Lung Screen Trial participants assessed reported low levels of PA and have a potentially modifiable risk factor to improve health outcomes. Larger studies are needed to characterize the burden of inactivity among high-risk lung cancer screening populations.

## Introduction

Physical activity (PA) has been implicated in the risk reduction of several cancers, including lung cancer.^1^ There have been systematic reviews evaluating the relationship between PA and lung cancer^2^ which revealed an inverse relationship between PA levels and lung cancer risk in people with active smoking histories. Similar associations have not been found in people who have never smoked; however, the effect of PA remained relevant when adjusted for smoking intensity and duration.^2^ This could be potentially explained by the biological differences in lung tumorigenesis between people with an active tobacco exposure and people without.^3^

The WHO recommends adults (aged ≥ 18 y old) should do at least 150 to 300 minutes of moderate-intensity aerobic PA or at least 75 to 150 minutes of vigorous-intensity aerobic PA or an equivalent combination of moderate and vigorous PA intensity throughout the week.^4^ The WHO also recommends at least 2 days of muscle-strengthening activities and limiting the amount of sedentary time.^4^ Nevertheless, 25% of adults do not meet the recommended levels of PA globally,^4^ with the WHO emphasizing an urgent need to implement and invest in interventions aimed at increasing PA.

Lung cancer screening with low-dose computed tomography has growing evidence of disease-specific mortality benefit in high-risk populations with active tobacco exposure and is supported by several international guidelines.^5^ As countries implement national lung cancer screening programs, it is important to characterize the burden of potentially modifiable risk factors. We report the first study to prospectively evaluate PA levels in a high-risk population undergoing lung cancer screening.

## Materials and Methods

Participants at a single Australian International Lung Screen Trial (ILST; NCT02871856) center were invited to participate in the PA observational study during usual ILST visits or follow-up contact. The ILST protocol has been published.^6^ In summary, the ILST recruited women and men aged 55 to 80 years old, with an active smoking history, and either an estimated 6-year lung cancer risk of more than or equal to 1.51% based on the PLCOm2012 risk prediction model or more than or equal to 30 pack-year smoking history, with a Eastern Cooperative Oncology Group performance status of 0 or 1. Participants were excluded from the PA observational study if they were not enrolled at the ILST site.

Outcomes were evaluated at a single time point as per study protocol. Self-reported outcomes were measured using the International Physical Activity Questionnaire (IPAQ),^7^ Physical Activity Scale for the Elderly (PASE)^8^ and EuroQol five-dimension questionnaires (EQ-5D-5L).^9^ The IPAQ requires participants to report their hours of PA from the last 7 days according to type (vigorous, moderate, walking, and sitting).^7^ These hours are then converted to Metabolic Equivalent Task (MET)/min/wk using the IPAQ formula. The PASE requires participants to report their leisure, household, and work-related activities in the last 7 days.^8^ PA levels were measured using a pedometer and 7-day step count diary. High PA levels are defined as more than or equal to 1500 MET/min/wk and moderate PA levels are more than or equal to 600 MET/min/wk on IPAQ. Furthermore, 150 minutes of moderate-intensity PA per week is equivalent to 600 MET/min/wk. Exercise capacity was measured with the 6-minute walk distance (6MWD).^10^ Hand grip muscle strength was measured using a dynamometer, with the highest value generated after three consecutive attempts for the database. There were five-second rests in between each repetition. Body composition was assessed with the InBody770 body composition analyzer (InBody USA, Cerritos, CA).^11^ Demographic and medical data were obtained including age, sex, smoking history, respiratory function, body mass index, working status, and education level. Study assessments were completed between 2019 and 2022; however, due to local coronavirus disease 2019 pandemic restrictions, in-person outcome measures occurring between 2020 and 2021 such as grip strength, 6MWD, and body composition had reduced completion rates (89%, 83%, and 49%, respectively). Data analysis was performed in R version 4.2.2.^12^ Parametric data are presented as mean and SD and nonparametric data are presented as median and interquartile range. Comparative analyses were performed using Kruskal-Wallis rank sum test for more than two independent variables, Pearson’s chi-square test, and Fisher’s exact test for categorical data. A *p* value of less than 0.05 was considered significant. The study was approved by the Melbourne Health Human Research Ethics Committee (HREC2018.401). Written informed consent was obtained from all participants.

## Results

A total of 178 participants of the 408 Royal Melbourne Hospital ILST participants were enrolled in the study. Participant characteristics of those enrolled and not enrolled are summarized in Table 1. Overall, the enrolled PA participants were representatives of the screening cohort. PA participants had a slightly lower PLCOm2012 score; however, there were no difference in smoking status or pack years and no significant difference on other characteristics except for percent-predicted forced expiratory volume in 1 second. There was a statistically significant lower forced expiratory volume in 1 second in those not enrolled; however, this was not clinically significant. Key physical activity and patient-reported and physical assessment outcomes are summarized in Table 2. Additional EQ-5D-5L, IPAQ, and PASE results are presented in Supplementary Table 1.

**Table 1:** Participant Characteristics

Characteristic	Enrolled (n = 178)^a^	Not Enrolled (n = 230)^a^	*p* Value^b^
**Age, y**	61 (59, 68)	63 (59, 68)	0.5
**Sex**			0.6
**Female**	70 (39%)	97 (42%)	
**Male**	108 (61%)	133 (58%)	
**Smoking status**			0.2
**Current**	91 (51%)	132 (57%)	
**Former**	87 (49%)	96 (42%)	
**Missing**	0 (0%)	2 (0.9%)	
**Pack-year history**	43 (36, 56)	47 (36, 57)	0.3
**PLCOm2012 score**	2.7 (1.8, 4.3)	3.5 (2.0, 5.9)	0.002
**BMI**	26.9 (24.3, 30.2)	27 (23.9, 29.9)	0.7
**Education**			>0.9
**8th grade**	6 (3.4%)	13 (5.7%)	
**9th to 11th grade**	51 (29%)	60 (26%)	
**High school graduate**	37 (21%)	43 (19%)	
**Technical/vocational certificate**	23 (13%)	33 (14%)	
**Some college or university**	17 (9.6%)	26 (11%)	
**University graduate**	25 (14%)	29 (13%)	
**Postgraduate**	18 (10%)	24 (11%)	
**Unknown**	1	2	
**Comorbidities**			
**Asthma**	19 (12%)	30 (16%)	0.7
**COPD**	7 (4.6%)	20 (11%)	0.055
**Cardiac disease**	32 (21%)	39 (22%)	0.8
**Diabetes**	16 (11%)	19 (10%)	>0.9
**Peripheral vascular disease**	2 (1%)	1 (1%)	0.5
**Stroke**	3 (2%)	10 (6%)	0.093
**Spirometry**			
**Forced expiratory ratio (%)**	72 (66, 77)	72 (63, 76)	0.14
**FEV**_**1**_**percent predicted (%)**	96 (83, 108)	92 (76, 103)	0.013

BMI, body mass index; COPD, chronic obstructive pulmonary disease; FEV_1_, forced expiratory volume in 1 second.

a Median (IQR); n (%).

b Wilcoxon rank sum test; Pearson’s chi-square test; Fisher’s exact test.

**Table 2:** Summary of Results

Health-related quality of life (EQ-5D-5L)		
VAS	Median (IQR)	82 (75, 90)
Self-reported PA levels		
IPAQ—Total (MET/min/wk)	Median (IQR)	1756 (689, 4049)
PASE—Total score	Mean (SD)	160 (72)
PA levels—pedometer and exercise diary (steps per day), n = 101		
	Median (IQR)	7237 (5353, 10,038)
Exercise capacity (6MWD) (m), n = 147		
	Mean (SD)	545 (92)
Hand grip strength		
Males, n = 96		
Right (kg)	Mean (SD)	31 (8)
Left (kg)	Mean (SD)	29 (7)
Females, n = 62		
Right (kg)	Mean (SD)	20 (6)
Left (kg)	Mean (SD)	18 (6)
Body composition		
Males, n = 51		
Skeletal muscle mass (kg)	Mean (SD)	33.7 (4.8)
Body fat mass(kg)	Mean (SD)	27.3 (10.8)
BMI (kg/m^2^)	Mean (SD)	29 (4)
Body fat (%)	Mean (SD)	30 (8)
Visceral fat area (cm^2^)	Mean (SD)	135 (58)
Females, n = 36		
Skeletal muscle mass (kg)	Mean (SD)	23.8 (4.0)
Body fat mass(kg)	Mean (SD)	27.8 (11.1)
BMI (kg/m^2^)	Mean (SD)	28 (5)
Body fat (%)	Mean (SD)	37 (8)
Visceral fat area (cm^2^)	Mean (SD)	144 (62)

6MWD, 6-minute walk distance; BMI, body mass index; EQ-5D-5L, EuroQol five-dimension; IPAQ, Physical Activity Questionnaire; IQR, interquartile range; MET, metabolic equivalent task; PA, physical activity; PASE, Physical Activity Scale for the Elderly; VAS, visual analogue scale.

When the cohort was stratified by level of PA intensity based on IPAQ total MET/min/wk, those with low PA levels had a higher pack-year history compared with the moderate and high PA groups; however, there were no significant clinical difference in spirometry and no statistical difference on current smoking status or other variables as presented in Table 3 and Supplementary Table 2.

**Table 3:** Cohort by Physical Activity Level

Characteristic	Level of Physical Activity^a^			*p* Value^b^
	High, n = 91	Moderate, n = 37	Low, n = 40	
**Age, y**	62 (59, 68)	61 (59, 70)	64 (60, 69)	0.5
**Sex**				0.2
**Male**	54 (59%)	19 (51%)	29 (73%)	
**Female**	37 (41%)	18 (49%)	11 (28%)	
**Smoking status**				0.7
**Current**	43 (47%)	19 (51%)	22 (55%)	
**Former**	48 (53%)	18 (49%)	18 (45%)	
**Pack years**	42 (35, 54)	41 (34, 49)	47 (40, 77)	0.02
**PLCOm2012 score**	2.45 (1.72, 4.58)	2.65 (1.75, 4.21)	2.81 (2.13, 4.45)	0.7
**Spirometry**				
**Forced expiratory ratio (%)**	71 (65, 77)	70 (65, 78)	70 (65, 75)	0.7
**FEV**_**1**_**percent predicted (%)**	95 (86, 109)	103 (92, 115)	98 (82, 107)	0.075
**FVC percent predicted (%)**	109 (101, 120)	116 (107, 128)	110 (100, 118)	0.051
**DLCO percent predicted (%)**	78 (69, 91)	80 (70, 96)	83 (74, 91)	0.5
**BMI**	27.0 (24.0, 30.0)	27.2 (24.0, 31.0)	28.4 (26.4, 33.0)	0.059
**EQ-5D-5L VAS**	85 (75, 90)	80 (73, 93)	80 (70, 90)	0.7
**Comorbidities**				
**Asthma**	9 (10%)	4 (11%)	5 (13%)	>0.9
**COPD**	4 (4%)	2 (5%)	1 (3%)	>0.9
**Cardiac disease**	17 (19%)	5 (14%)	9 (23%)	0.6
**Diabetes**	7 (8%)	5 (14%)	3 (8%)	0.5
**Peripheral vascular disease**	0 (0%)	1 (3%)	1 (3%)	0.2
**Stroke**	1 (1%)	1 (3%)	1 (3%)	0.8

BMI, body mass index; COPD, chronic obstructive pulmonary disease; DLCO, xxx; EQ-5D-5L, EuroQol five-dimension; FEV_1_, forced expiratory volume in 1 second; FVC, forced vital capacity; IQR, interquartile range; VAS, visual analogue scale.

a Median (IQR); n (%).

b Kruskal-Wallis rank sum test; Pearson’s chi-square test; Fisher’s exact test.

## Discussion

Of the cohort, 24% who completed the IPAQ reported low levels of PA and were not meeting guideline recommendations. Participants with low PA levels had a higher pack-year smoking history compared with those with moderate and high PA levels; however, there was no difference in smoking status (current or former) and spirometry. There was no difference in education level, working status, or comorbidities. Furthermore, 22% reported moderate PA levels and 54% reported high PA levels. This was consistent with PASE responses where the median total score was 160. This was higher than the average score reported for healthy but sedentary individuals aged 55 to 64 years (mean score 144, SD 76) and 65 years and older (mean score 119, SD 64).^8^ Median step count was in keeping with the average daily step count for Australian adults (7400 steps).^13^ Median 6MWD was within expected range for healthy adults (400 to 700 m).^14^ Median grip strength for men and women were also similar to optimal grip strengths of healthy adults.^15^ Only 51% of the cohort completed body composition. Body composition analysis revealed an elevated median body mass index, visceral fat area and percentage body fat, and low median skeletal muscle mass.^16^

Our understanding of the relationship between PA and lung cancer is evolving. A pooled study of seven Australian cohort studies estimated that if causal, PA levels below Australian guideline recommendations contributed to 15.6% (95% confidence interval: 6.9% to 23.4%) of lung cancers.^17^ In turn, lung cancer can have a negative impact on HRQoL and physical function.

Overall, our cohort of lung cancer screening participants who are at high risk for lung cancer based on the PLCOm2012 and smoking criteria has a reasonable level of PA compared with the general Australian population.^13^ This may be the result of selection bias in screening studies which results in a more proactive and health-conscious sample (healthy volunteer bias). Limitations of this study include its small sample size and the potential impacts of self-reporting. Nevertheless, balance of self-reporting was attempted by physical measurements. Owing to institutional interruptions during the coronavirus disease 2019 pandemic, body composition, grip strength, and 6MWD had lower completion rates. Sample size was affected by study design, where opportunities to limit multiple hospital attendances were prioritized, and participants were predominantly invited at the time the ILST scans and thus not all site participants were invited.

This is the first study to describe PA levels in a high-risk cohort of lung cancer screening participants. We identified a proportion (24%) of participants who reported low PA levels and participants who had a lower-than-expected exercise capacity and grip strength. In people who are already at high risk of lung cancer, PA levels may be a potentially modifiable risk factor. Screening is a recognized opportunity for intervention, with many lung cancer screening programs incorporating smoking cessation support. Larger studies are required to further characterize the burden of physical inactivity among lung cancer screening participants and to assess opportunity to intervene in the at-risk subpopulation.

## CRediT Authorship Contribution Statement

**Asha Bonney:** Investigation, Resources, Data Curation, Formal analysis, Writing—Original Draft, Writing—Review and Editing, Visualization, Project administration, Funding acquisition.

**Catherine L. Granger:** Methodology, Writing—Review and Editing, Visualization, Supervision.

**Daniel Steinfort:** Resources, Writing—Review and Editing, Supervision.

**Henry M. Marshall:** Conceptualization, Writing—Review and Editing, Supervision.

**Emily Stone:** Writing—Review and Editing.

**Annette McWilliams:** Writing—Review and Editing.

**Fraser Brims:** Writing—Review and Editing.

**Paul Fogarty:** Writing—Review and Editing.

**Linda Lin:** Data Curation, Writing—Review and Editing.

**Jiashi Li:** Data Curation, Writing—Review and Editing.

**Siyuan Pang:** Data Curation, Writing—Review and Editing.

**Stephen Lam:** Conceptualization, Methodology, Writing—Review and Editing.

**Kwun M. Fong:** Conceptualization, Methodology, Writing—Review and Editing, Supervision, Funding acquisition.

**Renee Manser:** Conceptualization, Methodology, Resources, Writing—Review and Editing, Visualization, Supervision, Funding acquisition.

## Disclosure

The authors declare no conflict of interest.
