# Supplementary material for: A Prospective Observational Study of Physical Activity Levels and Physical Fitness of People at High Risk for Lung Cancer
Source: JTO Clin Res Rep. 2024 Jan 13;5(2):100633. doi: 10.1016/j.jtocrr.2024.100633 (PMC10874747; doi:10.1016/j.jtocrr.2024.100633)
Supplement: Supplementary Tables [file mmc1.docx]

Table S1. EQ-5D-5L, IPAQ, and PASE outcomes for the whole cohort.

| Health-related quality of life (EQ-5D-5L) | | |
| --- | --- | --- |
| Mobility | Median (IQR) | 1 (1,2) |
| Personal care | Median (IQR) | 1 (1,1) |
| Usual activity | Median (IQR) | 1 (1,2) |
| Pain | Median (IQR) | 2 (1,2) |
| Anxiety/Depression | Median (IQR) | 1 (1,2) |
| Visual analogue scale (VAS) | Median (IQR) | 82 (75, 90) |
| Self-reported PA levels | | |
| IPAQ – Vigorous (MET/Min/Week) | Median (IQR) | 0, (0, 1080) |
| IPAQ – Moderate (MET/Min/Week) | Median (IQR) | 30 (0, 915) |
| IPAQ – Light (MET/Min/Week) | Median (IQR) | 792 (297, 1436) |
| IPAQ – Total (MET/Min/Week) | Median (IQR) | 1756 (689, 4049) |
| PASE – Walking outside | Mean (SD) | 19 (17) |
| PASE – Light sport | Mean (SD) | 8 (19) |
| PASE – Moderate sport | Mean (SD) | 729 (13) |
| PASE – Strenuous sport | Mean (SD) | 1 (4) |
| PASE – Muscle endurance | Mean (SD) | 5 (14) |
| PASE – Light housework | Mean (SD) | 22 (8) |
| PASE – Heavy housework | Mean (SD) | 19 (10) |
| PASE – Home repairs | Mean (SD) | 6 (12) |
| PASE – Lawn work | Mean (SD) | 20 (18) |
| PASE – Gardening | Mean (SD) | 14 (9) |
| PASE – Caring | Mean (SD) | 10 (16) |
| PASE – Volunteering | Mean (SD) | 33 (51) |
| PASE – Total score | Mean (SD) | 160 (72) |

Table S2. education and working status of cohort by PA level.

| Characteristic | Level of physical activity ^1^ | | | p-value^2^ |
| --- | --- | --- | --- | --- |
|  | High, N=91 | Moderate, N=37 | Low, N=40 |  |
| Education |  |  |  |  |
| 8^th^ grade | 4 (4%) | 0 (0%) | 1 (3%) |  |
| 9^th^ to 11^th^ grade | 28 (31%) | 8 (22%) | 11 (28%) |  |
| High school graduate | 14 (15%) | 9 (24%) | 10 (25%) |  |
| Technical/vocational certificate | 14 (15%) | 5 (14%) | 3 (8%) |  |
| Some college/university | 10 (11%) | 4 (11%) | 3 (8%) |  |
| University graduate | 9 (10%) | 9 (24%) | 7 (18%) |  |
| Postgraduate | 12 (13%) | 1 (3%) | 5 (13%) |  |
| Unknown | 0 | 1 | 0 |  |
| Work |  |  |  | 0.2 |
| Working | 42 (46%) | 13 (35%) | 12 (30%) |  |
| Retired | 27 (30%) | 16 (43%) | 16 (40%) |  |
| Disabled | 1 (1%) | 0 (0%) | 2 (5%) |  |
| Other | 4 (4%) | 0 (0%) | 1 (3%) |  |
| Unemployed | 3 (3%) | 3 (8%) | 1 (3%) |  |
| Unknown | 14 | 5 | 8 |  |

1 Median (IQR); n (%)

2 Kruskal-Wallis rank sum test
